# A pediatric case of anti-GABA_B_R encephalitis: case report and literature review

**DOI:** 10.3389/fped.2025.1563323

**Published:** 2025-08-25

**Authors:** Junxian Fu, Kairu Jia, Tianxia Li, Zhidong Qiao, Yuexin Jia, Xiaofan Yang, Hao Pan, Lu Zhao, Peirong Li, Manjie Zan, Guanglu Yang

**Affiliations:** ^1^ Department of Pediatrics, Affiliated Hospital of Inner Mongolia Medical University, Hohhot, Inner Mongolia, China; ^2^ Graduate School, Inner Mongolia Medical University, Hohhot, Inner Mongolia, China

**Keywords:** anti-GABABR encephalitis, children, autoimmune encephalitis, borderline encephalitis, clinical characteristics

## Abstract

**Background and objective:**

This study aims to analyze the clinical characteristics of anti-GABA_B_R encephalitis in pediatric patients. Due to its rarity and diagnostic challenges in children, we compare clinical features between adult and pediatric cases.

**Materials and methods:**

Using the key words “anti-GABA_B_R encephalitis, children, autoimmune encephalitis, limbic encephalitis”, we conduct a comprehensive literature review of all studies related to anti-GABA_B_R encephalitis published from January 2010 to January 2024. A total of 207 cases are identified globally, including 14 pediatric cases.

**Results:**

We report a case of an 8-year-and-6-month-old child with anti-GABA_B_R encephalitis presenting with abnormal mental behavior (irritability, hallucinations), sleep disorders, and paroxysmal involuntary limb movements. Serum anti-GABA_B_R antibodies were positive, and clinical symptoms improved significantly after corticosteroid treatment. Analysis reveal that children presented with mental/behavioral abnormalities as the initial symptom (85.71%), while adults presented with epileptic seizures as the initial symptom (76.71%). Main symptoms include epilepsy in adults (78.24%) and sleep disorders (26.67%) and involuntary limb movements (33.33%) in children. Neuroimaging shows higher involvement of the basal ganglia (55.56%), cerebellar hemispheres (22.22%), and brainstem (22.22%) in children compared to adults. Video electroencephalogram (EEG) analysis indicates more frequent abnormal EEG in adults, but epileptic waves are more common in children with abnormal EEG. Cerebrospinal fluid (CSF) cytology is not specific, with mild lymphocytic increases (adults 57.98% vs. children 33.33%, *P* = 0.2054). Despite higher prodromal fever rates in children (66.67% vs. 23.44%, *P* = 0.0228), they respond better to immunotherapy. No tumor-related issues are observed in pediatric cases, contrasting with 58.09% tumor comorbidity in adults.

**Conclusions:**

This study suggests that the clinical phenotypes of anti-GABABR encephalitis in children and adults may differ: children are more likely to present with mental and behavioral abnormalities (the initial symptom trend), sleep disorders and involuntary movements (the main symptoms), and their brain imaging is more likely to involve regions such as the basal ganglia and brainstem, and they respond better to immunotherapy. Notably, due to the small sample size of pediatric cases (*n* = 15) compared to adult cases (*n* = 193), these comparative findings should be interpreted with caution despite the statistical significance indicated by *P*-values. However, long-term follow-up remains essential.

## 1 Introduction

Autoimmune encephalitis refers to a group of encephalitis conditions triggered by autoimmune reactions. Since the initial identification of autoimmune encephalitis, various forms have been recognized based on the specific antibodies involved. These antibodies target different neuronal antigens, leading to different clinical manifestations and usually responding to immunotherapy (1).

Since the first report of anti-N-methyl-D-aspartate receptor (NMDAR) encephalitis in 2007 (2), people's understanding of it has gradually become clear. It is the most common form of autoimmune encephalitis, affecting both children and adults. It is characterized by a broad range of symptoms, which often evolves in a characteristic multiphasic pattern. Early symptoms are frequently psychiatric in nature, including acute behavioral changes, agitation, hallucinations, and delusions. As the disease progresses, patients may develop seizures, dyskinesias, movement disorders, and autonomic instability. Further progression can result in a decreased level of consciousness and even coma if not recognized and treated promptly (3). The diagnosis of anti-NMDAR encephalitis relies on the detection of NMDAR antibodies in the cerebrospinal fluid (CSF) or serum, along with clinical and neuroimaging findings. The main treatment measures for anti-NMDAR encephalitis are immunotherapy and tumor resection, and commonly used first-line immunotherapy methods include corticosteroids, immunoglobulins, and plasma exchange (4).

Other forms of autoimmune encephalitis have also been identified, each associated with specific antibodies. Anti-leucine-rich glioma inactivated protein antibody (anti-LGI1) encephalitis is characterized by limbic symptoms, such as memory loss and seizures, and often presents with hyponatremia. Anti-alpha-amino-3-hydroxy-5-methyl-4-isoxazolpropionic acid receptor (anti-AMPAR) encephalitis typically manifests with seizures, status epilepticus, and psychiatric symptoms. The diagnosis of these conditions relies on the detection of the respective antibodies in the CSF or serum, along with clinical and neuroimaging findings. Similar to anti-NMDAR encephalitis, the treatment involves immunotherapy, with the choice of therapy depending on the severity of the disease and the patient's response to initial treatment (1).

Anti-GABA_B_R encephalitis, the focus of this study, is a relatively rare form of autoimmune encephalitis. It is characterized by seizures and psychiatric symptoms. GABA_B_R is crucial for synaptic plasticity, which is related to neurotransmitter transmission, learning, memory and cognitive functions. Therefore, memory impairment, cognitive impairment and other conditions may also occur (5). The diagnosis is confirmed by the presence of GABA_B_R antibodies in the CSF or serum, following the diagnostic criteria provided by Graus et al. (6). Neuroimaging may show abnormalities in the limbic system or other brain regions. Treatment typically involves immunotherapy, with corticosteroids and intravenous immunoglobulin (IVIG) being the first-line options. In some cases, second-line therapies such as rituximab or cyclophosphamide may be required.

As of January 2024, a total of 208 cases of anti- GABA_B_R encephalitis have been reported globally in the literature. Among these, 14 are pediatric cases and 193 are adult cases. The present study describes an additional pediatric case, bringing the total number of reported pediatric cases to 15. This study aims to provide a reference for early clinical diagnosis and treatment by describing this new case and reviewing previously reported cases, while analyzing the similarities and differences between pediatric and adult anti-GABA_B_R encephalitis.

## 2 Materials and methods

Data collection was conducted through searches in China's National Knowledge Infrastructure (CNKI) and the newly added PubMed search function. A total of 207 cases reported both domestically and internationally from January 2010 to January 2024 were collected. Combined with the cases included in this article, there were 15 children and 193 adults. The clinical characteristics of both the pediatric and adult groups were examined. Descriptive statistics, including mean ± standard deviation (x ± s) or median values, are used for continuous data, while categorical data are presented as rates or percentages. For group comparisons, either the chi-square test or Fisher's exact test was employed, with a significance level set at *α* = 0.05. Statistical significance is considered when *P* < 0.05.

## 3 Results

### 3.1 Case presentation

#### 3.1.1 Medical history

An 8.5-year-old female patient was admitted in December 2023 with chief complaints of “abnormal mental behavior for 6 months, headache and dizziness for 4 months, and episodic involuntary limb movements for 1 week”.

Mental Behavior Phase (6 months prior): Non-specific onset of inattention, declining academic performance, irritability, crying spells, aggressive behavior, and sleep disturbances (insomnia, nightmares). Symptoms were intermittent and untreated.

Neurological Symptom Phase (4 months prior): Bilateral temporoparietal paroxysmal headaches (1–2 h/episode) with tinnitus and dizziness, exacerbated by exertion and relieved by rest, occurring daily. Occasional auditory hallucinations (mosquito buzzing, factory noise). Worsening 2 weeks prior included self-harm, aggression, and delusions of persecution (claiming bullying by teachers/peers).

Motor Dysfunction Phase (1 week prior): Nocturnal paroxysmal twitching and numbness in the right lower limb, progressing to 2–3 daily episodes (1–2 h/episode), disrupting sleep. Right upper limb tremors developed 4 days before admission; no fever, rash, or altered consciousness.

#### 3.1.2 Personal, past medical, and family history

Full-term vaginal delivery (birth weight 2.35 kg), uncomplicated perinatal course. Normal growth/development; no prior illnesses. Negative family history for similar conditions.

#### 3.1.3 Laboratory tests

Normal results for complete blood/urine/stool analyses, liver/kidney function, myocardial enzymes, electrolytes, glucose, ketones, lactate, ammonia, ceruloplasmin, homocysteine, vitamin D, parathyroid hormone, and thyroid function. Negative for lupus antibodies, ANCA, and infectious pathogens (mycoplasma, tuberculosis, streptococcus).

#### 3.1.4 Other examination

Normal findings on echocardiography, gynecological ultrasound, chest/abdominal computerized tomography (CT), and urinary system imaging. Non-contrast medium magnetic resonance imaging (MRI) of the head showed no lesions. During the interictal period (when the patient did not exhibit involuntary limb movements), video electroencephalogram (EEG) monitoring revealed prominent spikes, spike-and-slow waves/sharp-and-slow waves predominantly in the frontal lobe area in front of the central sulcus during sleep. During the onset of symptoms (when the patient experienced involuntary limb movements), video EEG monitoring did not show epileptiform discharges.

#### 3.1.5 CSF examination

Routine: Clear/colorless, negative Pandy's test, cell count 4 mm^3^. Biochemistry: LDH 21 U/L, glucose 3.21 mmol/L, protein 0.240 g/L, IgG 18.2 mg/L, chloride 124.1 mmol/L, lactate 1.63 mmol/L. Pathology/Immunology: Negative bacterial/tuberculosis stains, cryptococcal ink stain, viral antibodies, and cultures. Normal inflammatory cytokines (IL-2/4/6/10, TNF-α, IFN-γ, IL-17A). Serum anti-GABA_B_R IgG positive (1:10); all other autoantibodies and the oligoclonal bands negative.

#### 3.1.6 Diagnosis, treatment and outcome

Based on the clinical symptoms and serological anti-anti-GABABR IgG Ab, the patient was diagnosed as anti-GABA_B_R encephalitis. We carried out treatment based on the 2022 Edition of the Expert Consensus on the Diagnosis and Treatment of Autoimmune Encephalitis in China (7). The diagnosis and treatment guidelines mention that first-line immunotherapy includes glucocorticoids, intravenous immunoglobulin, and plasma exchange. Therefore, methylprednisolone sodium succinate was given for treatment (dosage: 20 mg·kg^−1^·d^−1^), and omeprazole sodium, vitamin D, calcium, and potassium chloride sustained-release tablets were provided to prevent the side effects of methylprednisolone sodium succinate. Post-treatment improvements: resolved mood/sleep disturbances, no limb movements, normal EEG. Subsequently, the patient began to take prednisone as prescribed regularly and gradually reduced the dosage until the medication was discontinued six months after discharge; 6-month follow-up showed stable remission.

### 3.2 Literature review and comparative analysis

#### 3.2.1 General demographic characteristics

Among the 208 patients who meet the diagnostic criteria for anti-GABA_B_R encephalitis, males predominate. The male-to-female ratio is 1.84:1 (125:68) in adult patients, with a mean age of 56.79 ± 10.07 years (range: 18–84 years). In the pediatric group, the ratio is 1.5:1 (9:6), with a mean age of 8.64 ± 5.35 years (range: 1–16 years) (Table 1).

**Table 1:** General demographic characteristics.

Indicators	Adult group (*N* = 193)	Pediatric group (*N* = 15)
Age, year	56.79 ± 10.07	8.64 ± 5.35
Sex, male: female, *n*	125: 68	9:6

#### 3.2.2 Clinical manifestations

##### 3.2.2.1 Prodromal symptoms

Among all the patients, a total of 70 patients (64 adults and 6 children) are recorded with prodromal symptoms, 19 (27.14%) experience prodromal symptoms of fever (19/70); 7 (10%) presented with headache as the prodromal symptom (7/70); 3 (4.29%) have diarrhea as a prodromal symptom (3/70); and 5 (7.14%) have cough as the prodromal symptom (5/70) (Table 2).

**Table 2:** Comparison of clinical characteristics between adults and children with anti-GABA_B_R encephalitis.

Clinical feature	Adult group (*N* = 193)	Pediatric group (*N* = 15)	*P*
Prodromal fever	23.44% (15/64)	66.67% (4/6)	0.0228*
Initial epileptic seizure	76.71% (56/73)	0% (0/7)	0.0001*
Initial mental behavioral abnormol	5.48% (4/73)	85.71% (6/7)	<0.0001*
Main epileptic seizure	78.24% (151/193)	26.67% (4/15)	<0.0001*
Sleep disorders	4.15% (8/193)	26.67% (4/15)	0.0063*
Involuntary limb movements	1.55% (3/193)	33.33% (5/15)	<0.0001*

1. *P* < 0.05 indicates statistical significance; 2. Initial symptom data were missing in 128 cases, limiting the reliability of these comparisons; 3. The pediatric group sample size (*n* = 15) is significantly smaller than the adult group (*n* = 193). Even with significant *P*-values, results should be interpreted cautiously to avoid overgeneralization.

*Statistical significance.

In the adult group, febrile prodromal symptoms are present in 15 out of 64 cases, representing a prevalence rate of 23.44% (15/64). Headache occurs in 6 cases (9.38%, 6/64), while 2 cases of diarrhea, and 4 cases of cough, accounting for rates of 3.13% (2/64) and 6.25% (4/64), respectively. Among the children, four out of six have fever as a prodromal symptom, indicating a prevalence rate of 66.67% (4/6). One child experiences headache (16.67%, 1/6), one has diarrhea (16.67%, 1/6). Additionally, one patient exhibits cough (16.67%, 1/6).

Overall, fever was the most common prodromal symptom, with a higher proportion in children than in adults. Headache, diarrhea, and cough were less frequent overall, with similar patterns of age-related differences (Table 2).

##### 3.2.2.2 Initial symptom

Among all the patients, a total of 80 patients are recorded with initial symptoms, the initial symptoms include epileptic seizures in 70% (56/80), mental behavior abnormalities in 12.5% (10/80), cognitive impairment in 21.25% (17/80), and consciousness disturbances in 6.25% (5/80).

Among the adults, the initial symptoms include epileptic seizures in 76.71% (56/73), mental and behavioral abnormalities in 5.48% (4/73), cognitive disorders in 23.29% (17/73), and consciousness disorders in 5.48% (4/73). In the pediatric group, the initial symptoms are mental and behavioral abnormalities in 85.71% (6/7), consciousness disturbances in 14.29% (1/7), with no cases of epileptic seizures or cognitive impairment as the initial symptoms (0/7).

To sum up, epileptic seizures were the predominant initial symptom in adults, while mental and behavioral abnormalities were most common in children. Cognitive impairment was observed as an initial symptom in adults but not in children, and consciousness disturbances were rare in both groups (Table 2).

##### 3.2.2.3 Main clinical manifestations

Among the patients, the main clinical manifestations include seizures in 74.52% (155/208), abnormal mental behavior in 51.92% (108/208), cognitive disorders in 45.67% (95/208), sleep disorders in 5.77% (12/208), and consciousness disturbances in 21.63% (45/208). Language disorders account for 7.69% (16/208), hallucinations for 8.65% (18/208), involuntary limb movements for 3.85% (8/208), inattention for 0.96% (2/208), and headaches for 3.37% (7/208).

In the adult group, 78.24% (151/193) of patients experience seizures, 51.81% (100/193) have mental and behavioral abnormalities, 47.15% (91/193) have cognitive disorders, 4.15% (8/193) have sleep disorders, and 22.23% (43/193) have consciousness disturbances. Language disorders are observed in 7.77% (15/193), hallucinations in 7.77% (15/193), involuntary limb movements in 1.55% (3/193), inattention in 0.52% (1/193), and headaches in 0.52% (5/193). In the pediatric group, 26.67% (4/15) of patients experience epilepsy, 53.33% (8/15) have abnormal mental behavior, 26.67% (4/15) have cognitive disorders, 26.67% (4/15) have sleep disorders, 13.33% (2/15) have consciousness disturbances, and 6.67% (1/15) have language disorders. Hallucinations are observed in 20.00% (3/15), involuntary limb movements in 33.33% (5/15), inattention in 6.67% (1/15), and headaches in 13.33% (2/15).

The main clinical manifestations varied between adult and pediatric patients. Seizures were far more common in adults, while children showed higher rates of sleep disorders, involuntary limb movements, and hallucinations. Both groups exhibited mental and behavioral abnormalities, though cognitive disorders were more prevalent in adults. Other symptoms such as language disorders, inattention, and headaches were relatively rare in both groups, with slight differences in their occurrence rates across age groups (Table 2).

#### 3.2.3 Auxiliary inspection

##### 3.2.3.1 CSF examination

Lumbar puncture was performed in 131 patients (119 adults and 12 children), with assessments including CSF pressure, cell count, glucose, protein, chloride levels, and IgG. Overall, CSF parameters showed similar patterns between adults and children, with no statistically significant differences. Minor variations were observed—for example, adult patients had a slightly higher rate of increased cell count and elevated IgG, while pediatric cases showed no changes in chloride levels or IgG (Table 3).

**Table 3:** Cerebrospinal fluid examination results for adult and pediatric patient groups.

CSF examination parameters	Adult group (*N* = 119)	Pediatric group (*N* = 12)	*P*
Elevated CSF pressure (mmHg) (Ref: 70–180)	9.24% (11/119)	8.33% (1/12)	>0.9999
Elevated CSF cell count (cells/μl) (Ref: 0–5)	57.98% (70/119)	33.33% (4/12)	0.2054
Elevated CSF protein (mg/dl) (Ref: 15–45)	42.86% (51/119)	33.33% (4/12)	0.7603
Elevated CSF glucose (mg/dl) (Ref: 40–70)	4.20% (5/119)	8.33% (1/12)	0.4448
Reduced CSF chloride ion (mmol/L) (Ref: 120–130)	2.52% (3/119)	0% (0/12)	>0.9999
Elevated CSF immunoglobulin G (IgG) (mg/L) (Ref: 10–40)	10.92%% (13/119)	0% (0/12)	0.6079

##### 3.2.3.2 Specific antibody detection and clinical characteristics

Antibody testing (serum and/or CSF) was conducted in 145 patients (130 adults and 15 children), with 63 cases lacking specific antibody data in the literature. The distribution of antibody positivity patterns differed between adults and children: adults more frequently tested positive for antibodies in both serum and CSF, while children showed a higher rate of exclusive serum positivity. Overall, serum antibody positivity was common in both groups, though CSF positivity rates were lower in children compared to adults (Table 4).

**Table 4:** Combined antibody test results for adults and children.

Antibody positivity pattern	Adult group (*N* = 130)	Pediatric group (*N* = 15)
Both serum and CSF positive	95 (73.08%)	8 (53.33%)
Only serum positive	18 (13.85%)	6 (40.00%)
Only CSF positive	17 (13.08%)	1 (6.67%)

“Both Serum and CSF Positive” = antibodies detected in both samples; “Only Serum Positive”/“Only CSF Positive” = antibodies detected exclusively in one sample. Total serum positivity (only serum + both) and total CSF positivity (only CSF + both) can be derived by summing the respective categories.

##### 3.2.3.3 Imaging examination

Cranial MRI or CT was performed in most patients (75.48%), with approximately half showing abnormal signals (excluding bleeding or infarction); these abnormalities were typically nonspecific hyperintensities on T2 and FLAIR sequences. Imaging rates and abnormal signal frequencies differed slightly by age: all pediatric patients underwent imaging, with a higher proportion showing abnormalities compared to adults (Table 5).

**Table 5:** Frequency of brain region involvement in cranial imaging.

Brain region	Adult group (*N* = 64, abnormal cases)	Pediatric group (*N* = 9, abnormal cases)	*P*
Frontal lobe	10.94% (7/64)	11.11% (1/9)	>0.9999
Temporal lobe	53.13% (34/64)	22.22% (2/9)	0.1522
Parietal lobe	4 (6.25%)	1 (11.11%)	0.4924
Occipital lobe	2 (3.13%)	1 (11.11%)	0.3301
Thalamus	2 (3.13%)	2 (22.22%)	0.0717
Basal ganglia	1.56% (1/64)	55.56% (5/9)	<0.0001*
Cerebellar hemisphere	1.56% (1/64)	22.22% (2/9)	0.0384*
Brainstem	0% (0/64)	22.22% (2/9)	0.0137*
Cingulate gyrus	1 (1.56%)	0 (0.00%)	>0.9999

1. *P* < 0.05 indicates statistical significance; 2. The number of pediatric cases with abnormal imaging is only 9, compared to 64 in the adult group. The significant *P*-values require further clinical validation due to the large sample size discrepancy.

*Statistical significance.

Notably, the distribution of involved brain regions varied significantly between age groups. Pediatric cases more frequently showed involvement of the basal ganglia, cerebellar hemispheres, and brainstem, whereas these regions were rarely affected in adults (Table 5).

##### 3.2.3.4 Electroencephalogram

EEG examinations were conducted in over half of the patients, with most showing abnormal results. Adults had a higher rate of abnormal EEG findings compared to children, though the pattern of abnormalities differed: epileptiform discharges were far more common in children with abnormal EEGs, whereas adults more frequently exhibited slow background rhythms. These age-related differences in EEG results are detailed in Table 6.

**Table 6:** Abnormal electroencephalogram (EEG) findings.

EEG abnormality	Adult group (*N* = 129)	Pediatric group (*N* = 9)	*P*
Abnormal results	115 (89.15%)	5 (55.56%)	0.0459*
Slow background rhythms	42 (36.52%)	2 (40.00%)	>0.9999
Epileptiform waves	37 (32.17%)	4 (80.00%)	0.0459*

1. *P* < 0.05 indicates statistical significance; 2. Normal EEG reference: Symmetrical background rhythms (alpha/beta waves) without pathological slow waves or epileptiform discharges; 3. The pediatric group's EEG sample size (9 cases) is significantly smaller than the adult group's (129 cases). The stability of results requires support from larger samples.

*Statistical significance.

##### 3.2.3.5 Tumor screening

Tumor imaging screening was performed in over 70% of patients, with notable differences between age groups. Adult patients showed a high rate of tumor detection, with lung cancer (predominantly small cell lung cancer) being the most common. In contrast, no tumors were identified in the pediatric group despite a high screening rate.

#### 3.2.4 Therapy

Immunotherapy patterns differed between adults and children. Over 60% of patients overall received first-line immunotherapy, with children undergoing first-line treatment universally, compared to about 60% of adults. The most common first-line approaches included glucocorticoids, immunoglobulins, and their combination, with plasma exchange used more frequently in children. Second-line immunotherapy was administered to a small proportion of both groups (Tables 7, 8).

**Table 7:** Treatment for adult and child patient groups.

Treatment	Adult group	Pediatric group	*P*
First-line therapy	*n* = 118	*n* = 15	
Hormones	31 (26.27%)	2 (13.33%)	0.3560
Gamma globulin	25 (21.19%)	1 (6.67%)	0.3013
Hormone + Gamma globulin	52 (44.07%)	7 (46.67%)	>0.9999
Plasma exchange	7 (5.93%)	3 (20.00%)	0.0861
Second-line therapy	*n* = 13	*n* = 2	
Azathioprine	3 (23.08%)	0 (0.00%)	>0.9999
Rituximab	4 (30.77%)	1 (50.00%)	>0.9999
Cyclophosphamide	6 (46.15%)	0 (0.00%)	0.4857
Methotrexate	0 (0.00%)	1 (50.00%)	0.1333

**Table 8:** Clinical remission rate with first-line drugs in adult and pediatric patient groups.

Group	Hormones			Gamma globulin			Hormone + Gamma globulin		
	Number of patients	Remission number	Clinical remission rate	Number of patients	Remission number	Clinical remission rate	Number of patients	Remission number	Clinical remission rate
Adult group	31	11	35.48%	25	5	20.00%	52	13	25.00%
Pediatric group	2	2	100.00%	1	1	100.00%	7	5	71.73%
Total	33	13	39.39%	26	6	23.08%	59	18	30.51%

The clinical remission rate was quantified by a Modified Rankin Scale (mRS) Score of ≤2 at follow-up.

Clinical remission (defined as MRS ≤2 at follow-up) showed age-related variations in response to different immunotherapies: children generally had higher remission rates across treatment types, including monotherapy with glucocorticoids or immunoglobulins, and combination therapy, compared to adults (Tables 7, 8).

#### 3.2.5 Outcome

Treatment outcomes were analyzed in 128 patients (113 adults and 15 children), with variations observed between age groups. Overall, nearly half of the patients achieved full or significant recovery following immunotherapy, while a similar proportion showed partial recovery—though residual symptoms such as cognitive impairment, memory issues, and mental health problems were common in this subgroup. A smaller percentage of patients did not survive (Table 9).

**Table 9:** Treatment outcome of adult and pediatric patient groups.

Treatment outcome	Adult group (*N* = 113)	Pediatric group (*N* = 15)	*P*
Fully restore	41 (36.28%)	11 (73.33%)	
Partial recovery	54 (47.79%)	2 (13.33%)	
Residual cognitive impairment	7 (12.96%)	0 (0.00%)	<0.0001*
Memory impairment due to past events	18 (33.33%)	0 (0.00%)	0.1270
Residual psychiatric symptoms	5 (9.26%)	1 (6.67%)	0.5342
Residual epilepsy	4 (7.41%)	0 (0.00%)	>0.9999
Leftover dizziness	2 (3.70%)	0 (0.00%)	>0.9999
Residual hallucination	2 (3.70%)	0 (0.00%)	>0.9999
Physical activity disorder	0 (0.00%)	1 (6.67%)	0.1172
Death	18 (15.93%)	2 (13.33%)	>0.9999
Recurrence	8 (7.08%)	1 (6.67%)	>0.9999

1. *P* < 0.05 indicates statistical significance; 2. The total sample size of the pediatric group (15 cases) is much smaller than that of the adult group (113 cases). Significant differences in outcomes such as residual symptoms may be influenced by sample size and require cautious generalization.

*Statistical significance.

Notably, children had a higher rate of full or substantial recovery compared to adults, with fewer residual symptoms. In contrast, adults more frequently experienced persistent cognitive impairment, and the mortality rate was higher in adults than in children. Detailed breakdowns of outcomes and residual symptoms are provided in Table 9.

## 4 Discussion

### 4.1 Core clinical differences and pathophysiological mechanisms

Anti-GABA_B_R encephalitis demonstrates distinct clinical phenotypes between children and adults, likely rooted in developmental neuroanatomy and synaptic plasticity. In terms of the initial symptoms, the limited available data (80 cases, with 128 cases missing data) indicated an age-related trend: Epileptic seizures were more common in adult patients, while children mostly presented with behavioral abnormalities. However, due to the lack of detailed records of initial symptoms in approximately 61.5% (128/208) of the cases, the reliability of this trend is limited. It should be interpreted with caution and cannot be regarded as a definitive conclusion. In the analysis of main symptoms with relatively complete data, the proportions of sleep disorders and involuntary movements in pediatric patients were significantly higher than those in adult patients, and the incidence of epilepsy in adults was even higher (*P* < 0.0001). This divergence may relate to age-dependent GABA-B receptor distribution: pediatric cases exhibit higher involvement of basal ganglia (55.56%), cerebellar hemispheres (22.22%), and brainstem (22.22%) (Table 5), regions critical for motor coordination and inhibitory control, whereas adult limbic-cortical networks are more susceptible to seizure generation (8–10). Combined with the fact that febrile prodromes are more common in children (66.67% vs. 23.44% in adults, *P* = 0.0228), it suggests that pediatric cases may have a more obvious pre-immune activation prodromal period, and this characteristic deserves clinical attention.

Electrophysiologically, adults show higher rates of abnormal EEG (89.15% vs. 55.56%, *P* = 0.0459), but children with abnormal EEG display more frequent epileptiform waves (80.00% vs. 32.17%, *P* = 0.0459) (Table 6). This paradox may reflect developmental differences in neuronal network stability—children's immature circuits are prone to subclinical epileptiform discharges, while adults require more severe inhibition loss to manifest clinical seizures (10, 11).

### 4.2 Therapeutic response and prognostic influencing factors

Pediatric patients exhibit superior treatment outcomes, with 100% clinical remission rates using monotherapy (corticosteroids or gamma globulin) and 71.73% with combination therapy (Table 8). This efficacy contrasts with adults (23.08%–39.39% remission) and may relate to two key factors: (1) absence of tumor comorbidity in children (0% vs. 58.09% in adults, Table 9), eliminating paraneoplastic immune activation; (2) potentially reversible synaptic dysfunction in developing brains, as opposed to adult neurons with higher vulnerability to chronic inflammation (12, 13).

It is worth noting that in adult cases, there is one patient with small cell lung cancer who does not receive immunotherapy during chemotherapy (cisplatin + etoposide) (14). The follow-up lung imaging after 6 months shows that the tumor has completely disappeared. However, as no neurological assessment is conducted, we have no idea about the recovery of the patient's neurological symptoms. Therefore, we consider that when combined with tumors, tumor treatment and immunotherapy are sometimes in conflict. On the one hand, immunotherapy (such as glucocorticoids and rituximab) supposes the immune system, and the effect of myelosuppression or immunosuppression in tumor treatment (such as chemotherapy and radiotherapy) superimposes, increasing the risk of infection. On the other hand, some scholars have speculated that (13) immunosuppressive therapy may promote the metastasis of neuroendocrine tumors, which is presumed to be related to “inhibiting the anti-tumor immune response”.

It is worth noting that although the clinical remission rate of first-line treatment for children is relatively high, there were still 2 cases of children who received second-line treatment (Table 7). Among them, one child patient's symptoms worsened after using gamma globulin, and then improved after receiving high-dose methylprednisolone pulse therapy and infusion of rituximab (15). Long-term follow-up is essential, as 13.33% of children showed partial recovery with residual motor or psychiatric symptoms (Table 9).

### 4.3 Differential diagnosis with anti-NMDAR encephalitis

Regarding the main symptoms, anti-GABA_B_R encephalitis is less stereotypical compared to the representative psychomotor symptoms seen in anti-NMDAR encephalitis. Additionally, the clinical phenotype of anti-GABA_B_R encephalitis remains stable over time, whereas the clinical presentation of anti-NMDAR encephalitis evolves (16). In terms of tumors, anti-NMDAR encephalitis is often accompanied by ovarian teratoma in children (17, 18), while no tumors occur in the children of this study. In terms of EEG, epileptic waves are more common in children with anti-GABA_B_R encephalitis, while diffuse slow waves are predominant in children with anti-NMDAR encephalitis (19). The above-mentioned differences are helpful for rapid clinical differentiation.

### 4.4 Limitations and future perspectives

The primary limitations are the small pediatric sample size (*n* = 15) and retrospective design, which may bias rare symptom analysis (e.g., consciousness disorders). Notably, the large discrepancy in sample size between pediatric and adult groups (15 vs. 193) undermines the robustness of comparative statistical analyses, even when significant *P*-values are reported. Prospective multicenter studies with ≥50 pediatric cases are needed to validate immune mechanisms and optimize treatment algorithms, particularly regarding the role of combination therapy in severe cases (16).

## 5 Conclusion

This study suggests that the clinical phenotypes of anti-GABABR encephalitis in children and adults may differ: children are more likely to present with mental and behavioral abnormalities (the initial symptom trend), sleep disorders and involuntary movements (the main symptoms), and their brain imaging is more likely to involve regions such as the basal ganglia and brainstem, and they respond better to immunotherapy. Notably, due to the small pediatric sample size (*n* = 15) compared to adults (*n* = 193), these findings—despite statistically significant *P*-values—remain preliminary and require validation in larger pediatric cohorts. It should be noted that the conclusion regarding the initial symptoms is limited by the lack of data. The overall conclusion still requires verification through studies with larger sample sizes and more complete data. The current findings can provide a reference for the identification and diagnosis of pediatric cases in clinical practice, but they cannot be used as an absolute diagnostic basis.

## Data Availability

The original contributions presented in the study are included in the article/Supplementary Material, further inquiries can be directed to the corresponding authors.
